# Assessing the risk of venous thromboembolism (VTE) in ambulatory patients with cancer: Rationale and implementation of a pharmacist-led VTE risk assessment program in an ambulatory cancer centre

**DOI:** 10.1177/10781552211004705

**Published:** 2021-03-24

**Authors:** Ryan Pelletier

**Affiliations:** School of Pharmacy, Faculty of Science, University of Waterloo, Kitchener, ON, Canada

**Keywords:** Risk prediction models, venous thromboembolism, ambulatory care, anticoagulation

## Abstract

**Objectives:**

The objectives of this paper were to identify and compare clinical prediction models used to assess the risk of venous thromboembolism (VTE) in ambulatory patients with cancer, as well as review the rationale and implementation of a pharmacist-led VTE screening program using the Khorana Risk Score model in an ambulatory oncology centre in Sault Ste. Marie, Ontario, Canada.

**Data Sources:**

PubMed was used to identify clinical practice guidelines and review articles discussing risk prediction models used to assess VTE risk in ambulatory patients with cancer.

**Data Summary:**

Three commonly used VTE risk prediction models in ambulatory patients with cancer: the Khorana Risk Score, Vienna Cancer and Thrombosis Study (CATS) and Protecht Score, were identified via literature review. After considering guideline recommendations, site-specific factors (i.e. laboratory costs, time pharmacists spent calculating VTE risk) and evidence from the CASSINI and AVERT trials, a novel pharmacist-led VTE risk assessment program using the Khorana Risk Score was developed during a fourth-year PharmD clinical rotation at the Algoma District Cancer Program (ADCP) [ambulatory cancer care centre]. ADCP patients with a Khorana Risk Score of ≥2 were referred to the hematologist for a full VTE workup. Considering limitations, inclusion and exclusion criteria of the CASSINI and AVERT trials, the hematologist and pharmacy team decided on appropriate initiation of thromboprophylaxis with a direct oral anticoagulant (DOAC).

**Conclusions:**

The Khorana Risk Score was the chosen model used for the pharmacist-led VTE risk assessment program due to its user-friendly scoring algorithm, evidence from validation studies and clinical trials, as well as ease of integration into pharmacy workflow. More research is needed to determine if pharmacist-led VTE risk assessment programs will impact patient outcomes, such as morbidity and mortality, secondary to cancer-associated thrombosis.

## Introduction

Patients with cancer are estimated to have a four to seven-fold increased risk of experiencing venous thromboembolism (VTE) compared to patients without cancer.^1^ Cancer induces a hypercoagulable state in affected patients via several pathophysiologic mechanisms beyond the scope of this practice tool.^2,3^ Additionally, patient risk factors including comorbid medical conditions (e.g. atrial fibrillation, diabetes mellitus) and low mobility may further exacerbate VTE risk.^4^ Cancer-associated thrombosis also confer increased morbidity (i.e. stroke, myocardial infarction) and mortality risks.^5^ In a prospective observational study of 4,466 patients with cancer receiving chemotherapy, thrombosis was the leading contributor of non-cancer causes of death in addition to infection (9.2% for each). Additionally, a staggering 47-fold increase in VTE death rate was noted in this study compared to the general population (VTE death rate reported was 448 per 1,00,000 patients, 95% confidence interval 6-89%, p = 0.03).^6,7^ Furthermore, cancer-associated thrombosis place added stress on government healthcare spending secondary to thrombotic complications that require urgent intervention and patient hospitalization (i.e. pulmonary embolism).^8^

Several risk prediction models have been developed to help clinicians assess VTE risk in ambulatory patients with cancer. Among the most widely recognized and used risk prediction models include the Khorana Score, Vienna Cancer and Thrombosis Study (CATS) Score and the Protecht Score.^9–11^ Each risk prediction model uses a combination of laboratory parameters (i.e. leukocyte count, platelet count), patient-specific factors (i.e. body mass index [BMI]), as well as chemotherapy-specific factors to calculate the percentage risk of developing VTE over a three to six-month period. The aims of this work are to summarize the intricacies of each risk prediction model mentioned above, as well as review the rationale and implementation of a pharmacist-led VTE screening program using the Khorana Risk Score model in an ambulatory oncology centre in Sault Ste. Marie, Ontario, Canada.

## Methods

A pharmacist-led VTE risk assessment program was developed during a fourth-year PharmD clinical rotation at ADCP in Sault Ste. Marie, Ontario, Canada. In the summer of 2020, ADCP pharmacists and a fourth-year PharmD clinical rotation student approached the program oncologists and hematologist with the idea of a pharmacist-led referral program for VTE risk assessment. The pharmacists, oncologists and hematologist agreed that a literature review be conducted to identify prediction models used to assess VTE risk in ambulatory patients with cancer. Risk prediction models were identified based on models reported in clinical practice guidelines,^12–17^ review articles^18^ and author knowledge. A PubMed search (inclusive of publications from inception until December 5, 2020) was conducted to identify systematic reviews discussing venous thromboembolism risk models used in ambulatory patients with cancer (search strategy: (“venous thromboembolism”[MeSH Terms]) AND (“risk”[MeSH Terms] OR “risk”[All Fields]) AND (“ambulatory care facilities”[MeSH Terms] OR “ambulatory”[All Fields]) AND (“neoplasms”[MeSH Terms] OR “cancer”[All Fields]) AND Review[ptyp]). Qualitative and quantitative data were extracted from review articles and primary literature identified via hand-searching reference lists. Qualitative data regarding the specific variables of each risk prediction model were extracted, including very high-risk tumor types, high-risk tumor types, presence of platinum chemotherapy and presence of gemcitabine chemotherapy. Patient inclusion and exclusion criteria were also extracted. Furthermore, quantitative data including reported three and six-month VTE rates, risk prediction model scoring algorithms and threshold laboratory values associated with each risk model scoring system were extracted.

Upon completion of the literature review and identification of relevant VTE risk prediction models, the pharmacists and clinical rotation student reviewed site-specific factors that may impact the implementation of identified models into ADCP pharmacy workflow. These site-specific factors included access to laboratory values required to calculate a score, as well as estimated time to complete a VTE risk assessment with each identified model. Once site-specific factors had been considered, ADCP pharmacists and clinical rotation student presented the oncologists and hematologist with a preferred VTE risk assessment model to be trialed in a pharmacist-led assessment program. The pharmacist-led assessment program implemented at ADCP consists of four steps: (1) calculation of the patient’s VTE risk score, (2) documentation of the score, as well as corresponding percentage risk and risk category in the patient’s chart, (3) referral of the patient to the clinical oncologist or hematologist for a full VTE workup, and (4) assessment of potential drug interactions and cost issues to optimize pharmacotherapy if an anticoagulant was initiated for the patient. VTE rates at ADCP were not available prior to program implementation.

## Results

Review of the literature consistently identified three commonly used risk prediction models that assess venous thromboembolism risk in ambulatory patients with cancer: (1) Khorana Risk Score, (2) Vienna CATS Score, and (3) Protecht Score.^9–11^ Six clinical practice guidelines identified via the search strategy were used to select the three risk prediction models: (1) National Comprehensive Cancer Network (NCCN), (2) American Society of Clinical Oncology (ASCO), (3) British Committee for Standards in Haematology, (4) International Initiative on Thrombosis and Cancer (ITAC), (5) European Society for Medical Oncology (ESMO), and (6) The Scientific Standards Committee (SSC) of the International Society of Thrombosis and Haemostasis (ISTH).^12–17^ Extracted data including the specific variables for each risk prediction model, risk prediction model scoring algorithms and threshold laboratory values associated with each risk model scoring system can be found in Table 1. VTE rates corresponding to each risk prediction model can be found in Table 2. Patient inclusion and exclusion criteria for each model are briefly discussed in text below.

**Table 1.** Venous thromboembolism (VTE) risk scoring comparison between Khorana, Vienna CATS, and Protecht clinical prediction models.

Risk variable	Khorana Risk Score (points)	Vienna CATS Score (points)	Protecht Score (points)
Very high-risk tumor (i.e. pancreatic or gastric)^a^	2	2	2
High-risk tumor (i.e. bladder, testicular, lymphoma, gynecological, lung)	1	1	1
Pre-chemotherapy platelet count ≥350 × 10 ^9^/L	1	1	1
Pre-chemotherapy leukocyte count ≥11 × 10 ^9^/L	1	1	1
Pre-chemotherapy hemoglobin <100 g/L or use of erythropoietin stimulating agents	1	1	1
Body mass index (BMI). ≥35 kg/m^2^	1	1	N/A
D-Dimer ≥35 mg/L	N/A	1	N/A
Soluble P-selectin ≥53.1 ng/L	N/A	1	N/A
Platinum chemotherapy	N/A	N/A	1
Gemcitabine chemotherapy	N/A	N/A	1

^a^Brain tumors are included in the ‘very high-risk’ tumor category for Vienna CATS Score calculation.

**Table 2.** Risk category stratification and percent risk of venous thromboembolism (VTE) for Khorana, Vienna CATS, and Protecht clinical prediction models.

Risk model	Risk Score	Risk category	Percent (%) VTE risk
Khorana Risk Score	0	Low	0.8% at 2.5 months^a^
	1–2	Intermediate	1.8% at 2.5 months
	≥3	High	7.1% at 2.5 months
Vienna CATS Score	0	Low	1.5% at 6 months
	1	Intermediate	3.8% at 6 months
	2	Intermediate	9.6% at 6 months
	≥3	High	17.7% at 6 months
Protecht Score	0–2	Low-intermediate	2% at 4 months
	≥3	High	8.1% at 4 months

^a^Data presented from Khorana Score derivation cohort.

### Khorana risk score

The Khorana Risk Score uses five readily available parameters to assign patients to low, intermediate, or high risk of VTE (Tables 1 and 2).^9^ It may be used to calculate VTE risk for patients with several solid tumor types and lymphomas; however, patients with myelomas and brain tumors were excluded from validation studies. Early recommendations suggested considering thromboprophylaxis for patients with a high-risk Khorana Score of ≥3 points .^19^ Randomized controlled trials have used the Khorana Risk Score as the VTE risk prediction model of choice in ambulatory patients with cancer. For example, both the CASSINI and AVERT randomized controlled trials used a Khorana Risk Score threshold of ≥2  (intermediate-to-high VTE risk) to consider patients eligible for thromboprophylaxis with DOACs. The CASSINI trial demonstrated a significantly decreased risk of VTE incidence in the secondary prespecified intervention-period analysis with rivaroxaban 10 mg once daily versus placebo, while the AVERT trial demonstrated a statistically significant decrease in VTE incidence with apixaban 2.5 mg twice daily versus placebo.^20,21^ Furthermore, the AVERT trial included patients with myelomas (2%) and brain tumors (5%), both of which were excluded from Khorana Score validation studies. The AVERT trial also reported a significant increase in major bleeding, while results from the CASSINI trial did not support this association.^20,21^ Although convenient, published literature has identified poor discriminatory ability between low and high-VTE risk patients for specific cancers using the Khorana Risk Score, such as pancreatic and lung.^22,23^

The Khorana Risk Score is the only independently validated VTE prediction tool that provides clinicians with a quick means of identifying ambulatory oncology patients that may benefit from the addition of thromboprophylaxis.^24^ The positive predictive value (probability of VTE in those deemed high-risk) of the Khorana Risk Score was found to be 7.1% and negative predative value (probability of not having VTE in those deemed low-risk) was found to be 98.5%. Sensitivity and specificity in the derivation cohort were found to be 40% and 88%, respectively.^9^ The Khorana Risk Score has also demonstrated benefit in identifying early VTE and predicting inpatient VTE-both of which are unknown with the Vienna CATS and Protecht Score models.^24^

### Vienna CATS score

The Vienna CATS Score introduced two biomarkers predictive of VTE risk in patients with cancer, D-Dimer and soluble P-selectin, in addition to the variables derived from the Khorana Risk Score (Tables 1 and 2).^10^ Patients with brain tumors were included in Vienna CATS model development and placed in the ‘very high risk’ cancer site category (i.e. 2 points). Furthermore, a patient’s VTE risk score would increase by 1 point if the D-Dimer was elevated ≥1.44 ug/mL , or if the soluble P-selection was elevated ≥51.3 ng/mL.  Patients were excluded if they had an overt bacterial or viral infection in the past two weeks, venous or arterial thromboembolism with the last three months, or continuous anticoagulation with vitamin K antagonists or low-molecular weight heparin (LMWH).^25^ The positive predictive value of the Vienna CATS Score was 22.1%, which was an improvement versus the Khorana Risk Score. The negative predictive value of the Vienna CATS score was reported as 94.9%, while the sensitivity was reported as 31.9% and specificity as 91.9%.^10^

A significant drawback of this model is the potential difficulty for laboratories to report D-dimer and soluble P-selectin as part of routine testing.^26^ From a practical standpoint, having to manually request these biomarkers may introduce disruptions to the pharmacist workflow via additional wait times for biomarker results to be reported. However, if D-Dimer and soluble P-selectin values are readily available as part of routine testing at a specific oncology centre, the Vienna CATS Score may be the preferred risk model as it has demonstrated superior positive predictive value of VTE compared to the Khorana Risk Score.^10^ The clinician should consider that evidence for thromboprophylaxis using the Vienna CATS Score employs injectable LMWH as the therapeutic intervention, while evidence for more convenient agents (i.e. direct oral anticoagulants) exists from the CASSINI and AVERT trials using the Khorana Score.^20,21^

### Protecht score

The Protecht Score investigators identified that specific chemotherapy agents, namely cisplatin, carboplatin and gemcitabine-based therapies, have a propensity to increase VTE risk in patients with cancer.^11^ This VTE risk prediction model employs the same variables included in the Khorana Risk Score, with the exception of BMI. Additionally, the Protecht Score adds an additional 1 point to the patient’s VTE risk score if the patient is on a platinum or gemcitabine-based chemotherapy (Tables 1 and 2). Several exclusion criteria were listed for the Protecht study, including but not limited to; confirmed arterial or venous thromboembolism in the past three months, antithrombic treatment for other indications, active bleeding in the past four weeks requiring hospitalization, and life expectancy of less than three months.^27^ Evidence from a prospective cohort study demonstrated superior discriminatory ability for low and high-VTE risk patients using the Protecht Score when compared to the Khorana Risk Score; however, it is still unclear whether the Protecht Score improves positive or negative predictive value of VTE versus the Khorana Risk Score.^24,26^ Positive and negative predictive values, as well as sensitivity and specificity data were not explicitly reported in the Protecht Score study.^11^

### Choice of model for Pharmacist-Led VTE risk assessment program

After considering evidence from the literature review and site-specific factors, the Khorana Risk Score was the model chosen to implement for the pharmacist-led VTE risk assessment program at ADCP. Site-specific factors for choosing the Khorana Risk Score included lack of timely and affordable access to soluble P-selectin laboratory values for all patients (therefore the Vienna CATS Score model was excluded), as well as time taken to complete the VTE risk assessment calculation. Via interview, ADCP pharmacists stated that the Khorana Risk Score calculation added approximately five minutes of working time for each patient assessed. The pharmacists did not feel that the addition of this task negatively impacted daily workflow. Evidence for the literature review also clearly indicated the Khorana Risk Score was the most validated tool among the three options, so this further influenced the decision to use the Khorana Risk Score model in practice versus the Protecht Score.^24^

## Discussion

### Guideline recommendations for VTE risk prediction models

Recommendations for the use of VTE risk prediction models in ambulatory patients with cancer vary among the guidelines identified. The NCCN guidelines identify the Khorana Risk Score and Vienna CATS Score models as potential tools that can be used to consider thromboprophylaxis in this patient population. However, the NCCN guidelines caution that broad use of these models in clinical practice should be avoided until efficacy of risk-adjusted thromboprophylaxis is demonstrated in randomized controlled trials.^12^ ASCO guidelines recommend that high-risk outpatients with cancer (Khorana Risk Score ≥ 2 or higher prior to chemotherapy) may be offered thromboprophylaxis with a DOAC (i.e. apixaban or rivaroxaban) or LMWH. Additionally, a risk-benefit discussion between prescriber and patient relaying cost and duration of therapy should be considered.^13^ Guidelines from the British Committee for Standards in Haematology briefly mentions the Khorana Risk Score as a tool that can be used to identify patient at high risk of thrombosis. The Vienna CATS or Protecht Score are not mentioned in this guideline.^14^

The ITAC guidelines state that the Khorana Risk Score is the most widely used model to identify ambulatory patients with cancer at high risk of thrombosis. These guidelines recommend prophylaxis with apixaban or rivaroxaban if the patient’s Khorana Risk Score is ≥2 and are not at high risk of, or actively bleeding. The Vienna CATS Score and Protecht Score are briefly mentioned as variations of the Khorana Risk Score that may improve risk assessment.^15^ The ESMO guidelines identify that the Khorana Risk Score may be used to identify ambulatory patients with cancer with are at clinically high risk for VTE. ESMO guidelines also identify limitations of the Khorana Risk Score, such as the exclusion of high-risk cancer types (e.g. brain cancer) from the study population.^16^ Furthermore, the SCC and ISTH guidelines suggest the use of DOACs (i.e. apixaban or rivaroxaban) as primary thromboprophylaxis for ambulatory patients with cancer if they have a Khorana Score ≥2 , have no drug interactions, and are not considered high risk for bleeding. SCC and ISTH guidelines also highlight the importance of shared decision-making and considering patient preferences when deciding to initiate thromboprophylaxis.^17^

### Integration of the Khorana risk score in clinical oncology practice

**Figure 1.**
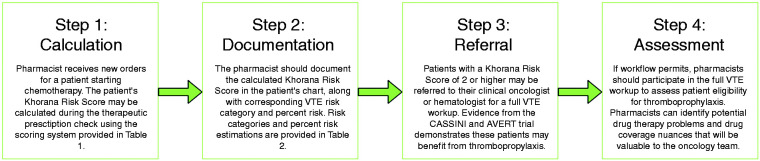
Proposed framework for pharmacist-initiated venous thromboembolism (VTE) risk prediction in patients with cancer using the Khorana Risk Score.

As some laboratories may have difficulty obtaining specific biomarkers (e.g. soluble P-selectin required to calculate Vienna CATS Score), the Khorana Risk Score is a convenient tool that uses readily accessible laboratory parameters available across most oncology practice sites. Therefore, pharmacists working in ambulatory clinical oncology centres may be appropriately positioned to incorporate the Khorana Risk Score into daily workflow. The pharmacist-led VTE assessment program implemented at ADCP is summarized in Figure 1. In only a few short moments, a Khorana Risk Score can be calculated for patients starting chemotherapy with many of the same parameters used to verify the therapeutic appropriateness of prescribed drug regimens. Once the Khorana Risk Score is calculated, pharmacists should document the calculated score and corresponding percentage VTE risk in the patient’s physical and/or electronic chart(s).

Using evidence from the CASSINI and AVERT randomized controlled trials, pharmacists may refer patients with a Khorana Risk Score ≥2 to the most responsible medical oncologist or hematologist for a complete VTE risk evaluation, as well as participate in the decision to initiate thromboprophylaxis or not.^20,21^ However, one must consider the limitations of these trials when deciding to recommend initiation of rivaroxaban or apixaban for VTE prophylaxis in ambulatory patients with cancer. In the CASSINI trial, nearly 47% of enrolled patients discontinued rivaroxaban early in the treatment group. Additionally, a significant VTE benefit was only observed in a secondary prespecified intervention-period analysis (2.6% intervention vs. 6.4% control; Hazard Ratio 0.40, 95% confidence interval 0.20-0.80), which is subject to bias. The primary outcome of VTE benefit did not reach statistical significance (6.0% intervention vs. 8.8% control; Hazard Ratio 0.66, 95% confidence interval 0.40-1.09, p = 0.10).^20^ In the AVERT trial, only 5.9% of patients had a creatinine clearance of 50 ml/min or less. Therefore, applicability of AVERT trial results to patients with a creatinine clearance of 50 ml/min or less is questioned.^21^ After consideration of trial limitations, pharmacists should also notify the oncologist or hematologist if the patient is already receiving anticoagulation or antiplatelet therapy for another indication. Navigating drug coverage options for patients that start thromboprophylaxis is another area in which both hospital and community pharmacists may significantly contribute to optimize patient care. Although pharmacists may not always make the final decision on initiating thromboprophylaxis for a referred patient, identifying those that may benefit from the addition of an anticoagulant is a crucial step in the process of minimizing preventable cancer-associated thrombosis.

A pharmacist-led model of VTE risk assessment using the Khorana Risk Score was implemented in the summer of 2020 during a fourth-year PharmD clinical rotation at the Algoma District Cancer Centre (ADCP) [Sault Area Hospital] in Sault Ste. Marie, Ontario, Canada. All new ambulatory patients with cancer, as well as patients with cancer recurrence, receiving treatment at ADCP were screened for VTE risk by the pharmacist while therapeutically assessing the first cycle of chemotherapy. If the patient’s Khorana Risk Score was ≥2 , the patient was referred to the hematologist for a full VTE workup to determine if thromboprophylaxis should be initiated. Once referred, the hematologist determined if the patient met CASSINI or AVERT inclusion criteria during the full VTE workup assessment. During the eight-week PharmD clinical rotation period, several ADCP patients were referred by the pharmacists and pharmacy student to the hematologist for a full VTE workup, particularly if the patients were not receiving indefinite thromboprophylaxis for another indication. The number of patients referred for full VTE workup was not tracked during this time; however, since pharmacist-led VTE assessment using the Khorana Score model has continued to be used by ADCP pharmacists after inception, an original investigation assessing quantitative patient outcomes (i.e. VTE rates pre and post-pharmacist referral program use) is currently in planning stages. The pharmacist-led referral system for full VTE workup could play a critical role in identifying patients who would benefit from starting thromboprophylaxis that otherwise may be overlooked at this site.

### Limitations

Despite the conveniences of using risk prediction models to assess VTE risk in ambulatory patients with cancer, limitations to these tools are evident. A patient’s risk of VTE is dynamic and changes over time; however, risk prediction models use parameters from a single laboratory panel to calculate a three to six-month risk of VTE.^28^ Risk prediction models are not a replacement for clinician judgement, and patient factors outside of the risk model variables (i.e. drug interactions, inherited clotting disorders, patient history of VTE and bleeding) must be considered when deciding to initiate, or not to initiate, thromboprophylaxis. Furthermore, limitations to implementing a pharmacist-led VTE risk assessment program at an ambulatory cancer centre exist. Although a Khorana Risk Score only takes minutes to calculate, some pharmacists may be reluctant to add this task to an already demanding workday. Additionally, patient drug coverage will vary substantially by geographic region, and the DOACs (i.e. rivaroxaban, apixaban) supported by evidence from the CASSINI and AVERT trials are significantly more expensive than alternative medications with demonstrated therapeutic benefit in preventing cancer-associated thrombosis (i.e. warfarin).^29^ However, it can be argued that patients may make up for this medication cost difference in both time and money saved not having to monitor the INR. Furthermore, warfarin is not considered a first-line agent in VTE prophylaxis for ambulatory patients with cancer.^29^ Pharmacists, other clinicians and patients must continue to advocate for government or other third-party drug coverage that will allow greater accessibility to convenient medications, such as DOACs, with increasing evidence for this indication.

Limitations of this study are also evident. The three models identified in this study (i.e. Khorana Risk Score, Vienna CATS Score and Protecht Score) are not exhaustive of all prediction models used to assess VTE risk in ambulatory patients with cancer. However, these three models were the most consistently discussed in clinical practice guidelines and review articles identified in our search strategy. Although other models exist, the Khorana Risk Score still has the most robust external validation evidence to date.^24^ Furthermore, a notable limitation of this study is the lack of pre and post-intervention VTE rate, as well as patient-specific outcome data for the ADCP pharmacist-led VTE risk assessment program that is available at this current time. ADCP pharmacists have slowly incorporated this model into their workflow since the summer of 2020, and an original research investigation assessing patient outcomes using the pharmacist-led program is currently in development.

## Conclusions

Three risk prediction models used to assess VTE risk in ambulatory patients with cancer, the Khorana Risk Score, Vienna CATS Score, and the Protecht score were identified in this study. Given the Khorana Risk Score’s user-friendly algorithm and potential for integration into pharmacy workflow, a pharmacist-led VTE risk assessment program using the Khorana Risk Score was introduced at an ambulatory oncology centre in Sault Ste. Marie, Ontario to aid in identifying ambulatory oncology patients at risk of developing VTE that may benefit from thromboprophylaxis. Incorporating VTE risk assessment into pharmacist practice may foster interprofessional communication within the oncology care team, especially when deciding to initiate thromboprophylaxis in eligible patients. Further work is needed to determine if pharmacist-led VTE risk assessment will impact patient outcomes, such as morbidity and mortality, secondary to cancer-associated thrombosis.
